# Supplementary material for: Synergy Assessment of Four Antimicrobial Bioactive Compounds for the Combinational Treatment of Bacterial Pathogens
Source: Biomedicines. 2023 Aug 7;11(8):2216. doi: 10.3390/biomedicines11082216 (PMC10452528; doi:10.3390/biomedicines11082216)
Supplement: Supplementary file 1 [file biomedicines-11-02216-s001.zip › biomedicines-2505753-supplementary.pdf]

Supplementary Materials

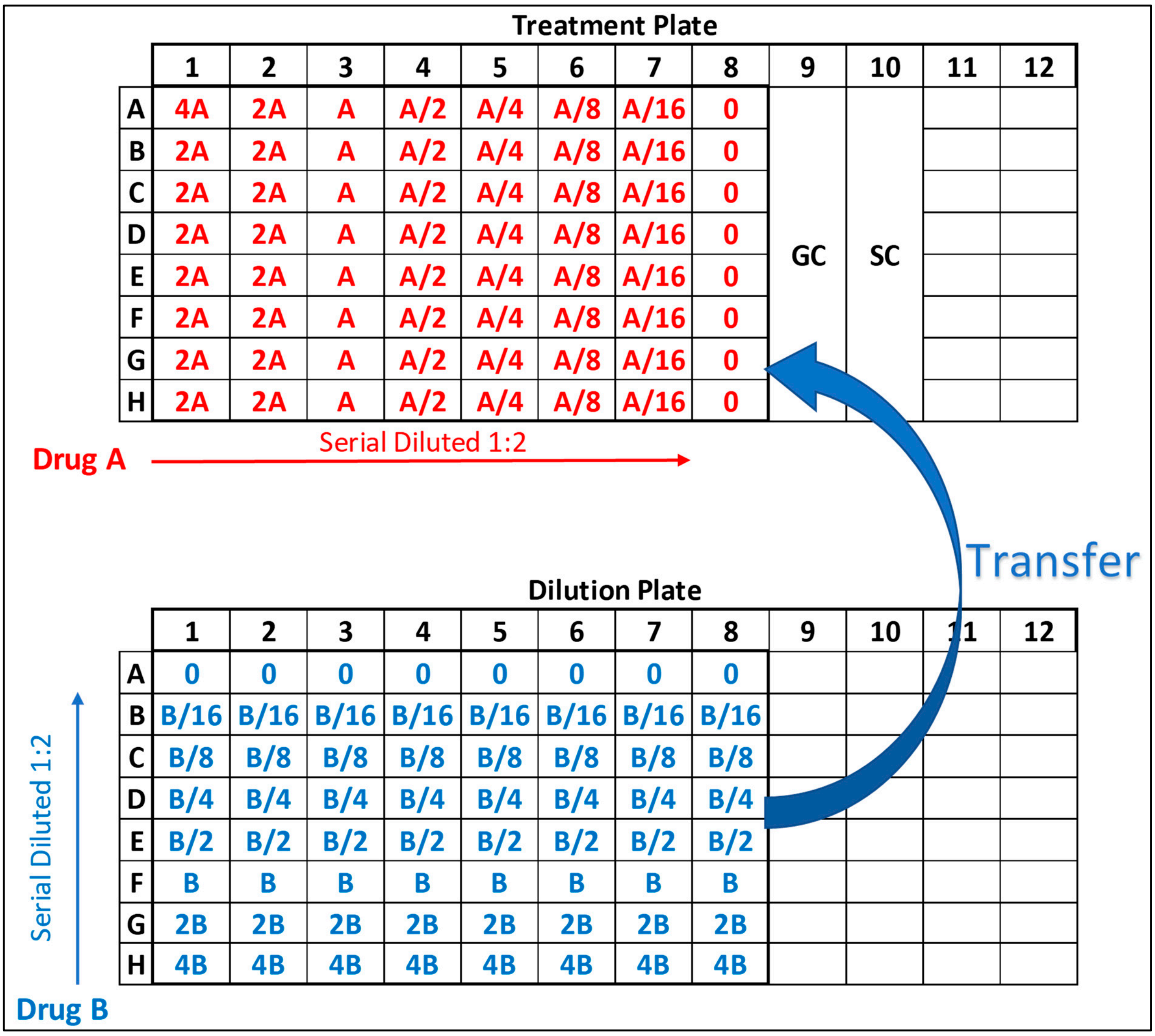

Figure S1. Drug Combination Set Up: Layout of Two-drug combination treatment plate and dilution plate before transfer step. **A** refers to concentration of Drug A, **B** refers to the concentration of Drug B, showing the dilution factor of each following the serial dilution. **GC**: Growth Control, **SC**: Sterility Control.

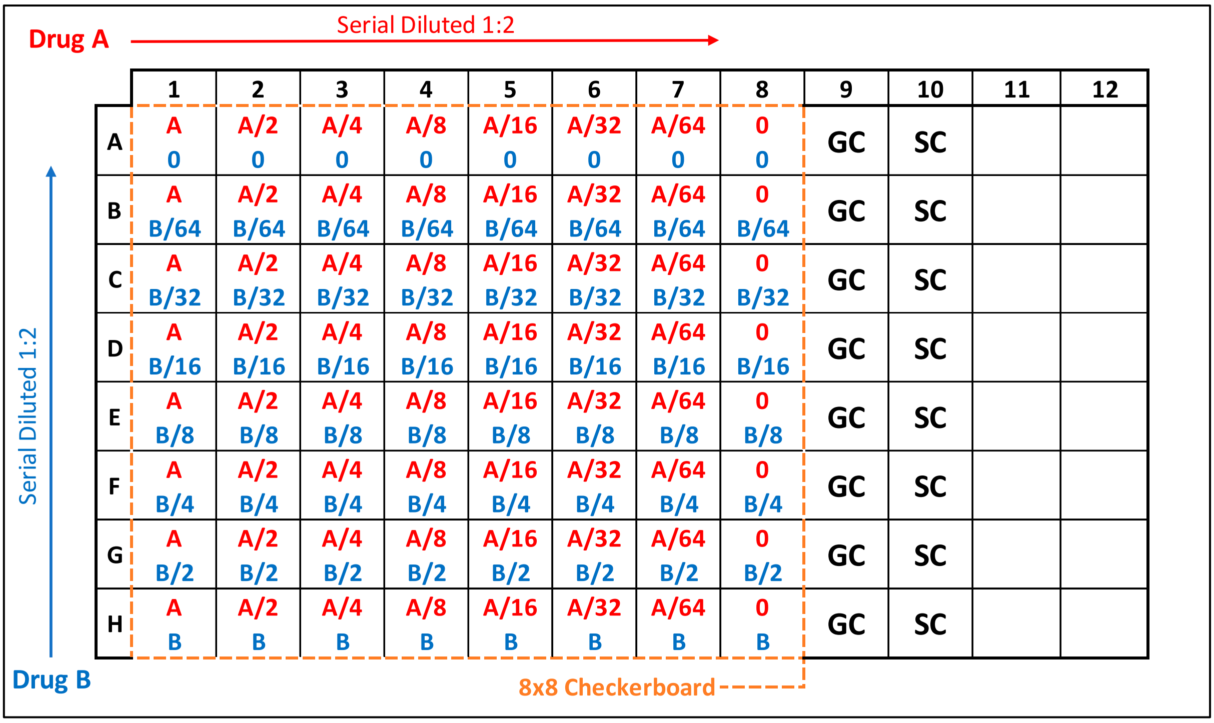

Figure S2. Two-Drug combination Assay Final Layout: Final layout of 96-well plate for a Two-Drug combination assay. **A** refers to concentration of Drug A, **B** refers to the concentration of Drug B, showing the dilution factor of each following the serial dilution, transfer step and addition of inoculum. The 8x8 checkerboard is labelled with dashed lines. **GC**: Growth Control, **SC**: Sterility Control.

Plate 1

6x6 Checkerboard — CB1 (columns 1–6, rows A–F); CB2 (columns 7–12, rows A–F)

	1	2	3	4	5	6	7	8	9	10	11	12
A	A<br>0<br>C	A/2<br>0<br>C	A/4<br>0<br>C	A/8<br>0<br>C	A/16<br>0<br>C	0<br>0<br>C	A<br>0<br>C/2	A/2<br>0<br>C/2	A/4<br>0<br>C/2	A/8<br>0<br>C/2	A/16<br>0<br>C/2	0<br>0<br>C/2
B	A<br>B/16<br>C	A/2<br>B/16<br>C	A/4<br>B/16<br>C	A/8<br>B/16<br>C	A/16<br>B/16<br>C	0<br>B/16<br>C	A<br>B/16<br>C/2	A/2<br>B/16<br>C/2	A/4<br>B/16<br>C/2	A/8<br>B/16<br>C/2	A/16<br>B/16<br>C/2	0<br>B/16<br>C/2
C	A<br>B/8<br>C	A/2<br>B/8<br>C	A/4<br>B/8<br>C	A/8<br>B/8<br>C	A/16<br>B/8<br>C	0<br>B/8<br>C	A<br>B/8<br>C/2	A/2<br>B/8<br>C/2	A/4<br>B/8<br>C/2	A/8<br>B/8<br>C/2	A/16<br>B/8<br>C/2	0<br>B/8<br>C/2
D	A<br>B/4<br>C	A/2<br>B/4<br>C	A/4<br>B/4<br>C	A/8<br>B/4<br>C	A/16<br>B/4<br>C	0<br>B/4<br>C	A<br>B/4<br>C/2	A/2<br>B/4<br>C/2	A/4<br>B/4<br>C/2	A/8<br>B/4<br>C/2	A/16<br>B/4<br>C/2	0<br>B/4<br>C/2
E	A<br>B/2<br>C	A/2<br>B/2<br>C	A/4<br>B/2<br>C	A/8<br>B/2<br>C	A/16<br>B/2<br>C	0<br>B/2<br>C	A<br>B/2<br>C/2	A/2<br>B/2<br>C/2	A/4<br>B/2<br>C/2	A/8<br>B/2<br>C/2	A/16<br>B/2<br>C/2	0<br>B/2<br>C/2
F	A<br>B<br>C	A/2<br>B<br>C	A/4<br>B<br>C	A/8<br>B<br>C	A/16<br>B<br>C	0<br>B<br>C	A<br>B<br>C/2	A/2<br>B<br>C/2	A/4<br>B<br>C/2	A/8<br>B<br>C/2	A/16<br>B<br>C/2	0<br>B<br>C/2
G	GC	GC	GC	GC	GC	GC	GC	GC	GC	GC	GC	GC
H	SC	SC	SC	SC	SC	SC	SC	SC	SC	SC	SC	SC

Plate 2

CB3 (columns 1–6, rows A–F); CB4 (columns 7–12, rows A–F)

	1	2	3	4	5	6	7	8	9	10	11	12
A	A<br>0<br>C/4	A/2<br>0<br>C/4	A/4<br>0<br>C/4	A/8<br>0<br>C/4	A/16<br>0<br>C/4	0<br>0<br>C/4	A<br>0<br>C/8	A/2<br>0<br>C/8	A/4<br>0<br>C/8	A/8<br>0<br>C/8	A/16<br>0<br>C/8	0<br>0<br>C/8
B	A<br>B/16<br>C/4	A/2<br>B/16<br>C/4	A/4<br>B/16<br>C/4	A/8<br>B/16<br>C/4	A/16<br>B/16<br>C/4	0<br>B/16<br>C/4	A<br>B/16<br>C/8	A/2<br>B/16<br>C/8	A/4<br>B/16<br>C/8	A/8<br>B/16<br>C/8	A/16<br>B/16<br>C/8	0<br>B/16<br>C/8
C	A<br>B/8<br>C/4	A/2<br>B/8<br>C/4	A/4<br>B/8<br>C/4	A/8<br>B/8<br>C/4	A/16<br>B/8<br>C/4	0<br>B/8<br>C/4	A<br>B/8<br>C/8	A/2<br>B/8<br>C/8	A/4<br>B/8<br>C/8	A/8<br>B/8<br>C/8	A/16<br>B/8<br>C/8	0<br>B/8<br>C/8
D	A<br>B/4<br>C/4	A/2<br>B/4<br>C/4	A/4<br>B/4<br>C/4	A/8<br>B/4<br>C/4	A/16<br>B/4<br>C/4	0<br>B/4<br>C/4	A<br>B/4<br>C/8	A/2<br>B/4<br>C/8	A/4<br>B/4<br>C/8	A/8<br>B/4<br>C/8	A/16<br>B/4<br>C/8	0<br>B/4<br>C/8
E	A<br>B/2<br>C/4	A/2<br>B/2<br>C/4	A/4<br>B/2<br>C/4	A/8<br>B/2<br>C/4	A/16<br>B/2<br>C/4	0<br>B/2<br>C/4	A<br>B/2<br>C/8	A/2<br>B/2<br>C/8	A/4<br>B/2<br>C/8	A/8<br>B/2<br>C/8	A/16<br>B/2<br>C/8	0<br>B/2<br>C/8
F	A<br>B<br>C/4	A/2<br>B<br>C/4	A/4<br>B<br>C/4	A/8<br>B<br>C/4	A/16<br>B<br>C/4	0<br>B<br>C/4	A<br>B<br>C/8	A/2<br>B<br>C/8	A/4<br>B<br>C/8	A/8<br>B<br>C/8	A/16<br>B<br>C/8	0<br>B<br>C/8
G	GC	GC	GC	GC	GC	GC	GC	GC	GC	GC	GC	GC
H	SC	SC	SC	SC	SC	SC	SC	SC	SC	SC	SC	SC

Plate 3

CB5 (columns 1–6, rows A–F); CB6 (columns 7–12, rows A–F)

	1	2	3	4	5	6	7	8	9	10	11	12
A	A<br>0<br>C/16	A/2<br>0<br>C/16	A/4<br>0<br>C/16	A/8<br>0<br>C/16	A/16<br>0<br>C/16	0<br>0<br>C/16	A<br>0<br>0	A/2<br>0<br>0	A/4<br>0<br>0	A/8<br>0<br>0	A/16<br>0<br>0	0<br>0<br>0
B	A<br>B/16<br>C/16	A/2<br>B/16<br>C/16	A/4<br>B/16<br>C/16	A/8<br>B/16<br>C/16	A/16<br>B/16<br>C/16	0<br>B/16<br>C/16	A<br>B/16<br>0	A/2<br>B/16<br>0	A/4<br>B/16<br>0	A/8<br>B/16<br>0	A/16<br>B/16<br>0	0<br>B/16<br>0
C	A<br>B/8<br>C/16	A/2<br>B/8<br>C/16	A/4<br>B/8<br>C/16	A/8<br>B/8<br>C/16	A/16<br>B/8<br>C/16	0<br>B/8<br>C/16	A<br>B/8<br>0	A/2<br>B/8<br>0	A/4<br>B/8<br>0	A/8<br>B/8<br>0	A/16<br>B/8<br>0	0<br>B/8<br>0
D	A<br>B/4<br>C/16	A/2<br>B/4<br>C/16	A/4<br>B/4<br>C/16	A/8<br>B/4<br>C/16	A/16<br>B/4<br>C/16	0<br>B/4<br>C/16	A<br>B/4<br>0	A/2<br>B/4<br>0	A/4<br>B/4<br>0	A/8<br>B/4<br>0	A/16<br>B/4<br>0	0<br>B/4<br>0
E	A<br>B/2<br>C/16	A/2<br>B/2<br>C/16	A/4<br>B/2<br>C/16	A/8<br>B/2<br>C/16	A/16<br>B/2<br>C/16	0<br>B/2<br>C/16	A<br>B/2<br>0	A/2<br>B/2<br>0	A/4<br>B/2<br>0	A/8<br>B/2<br>0	A/16<br>B/2<br>0	0<br>B/2<br>0
F	A<br>B<br>C/16	A/2<br>B<br>C/16	A/4<br>B<br>C/16	A/8<br>B<br>C/16	A/16<br>B<br>C/16	0<br>B<br>C/16	A<br>B<br>0	A/2<br>B<br>0	A/4<br>B<br>0	A/8<br>B<br>0	A/16<br>B<br>0	0<br>B<br>0
G	GC	GC	GC	GC	GC	GC	GC	GC	GC	GC	GC	GC
H	SC	SC	SC	SC	SC	SC	SC	SC	SC	SC	SC	SC

Figure S3. Three Drug combination Assay Final Layout: Final 96-well plate layout for a Three-Drug combination assay. **A** refers to concentration of Drug A, **B** refers to the concentration of Drug B, **C** refers to the concentration of Drug C, showing the dilution factor of each following the serial dilution, transfer steps and addition of inoculum. **CB**: Checkerboard, **GC**: Growth Control, **SC**: Sterility Control.

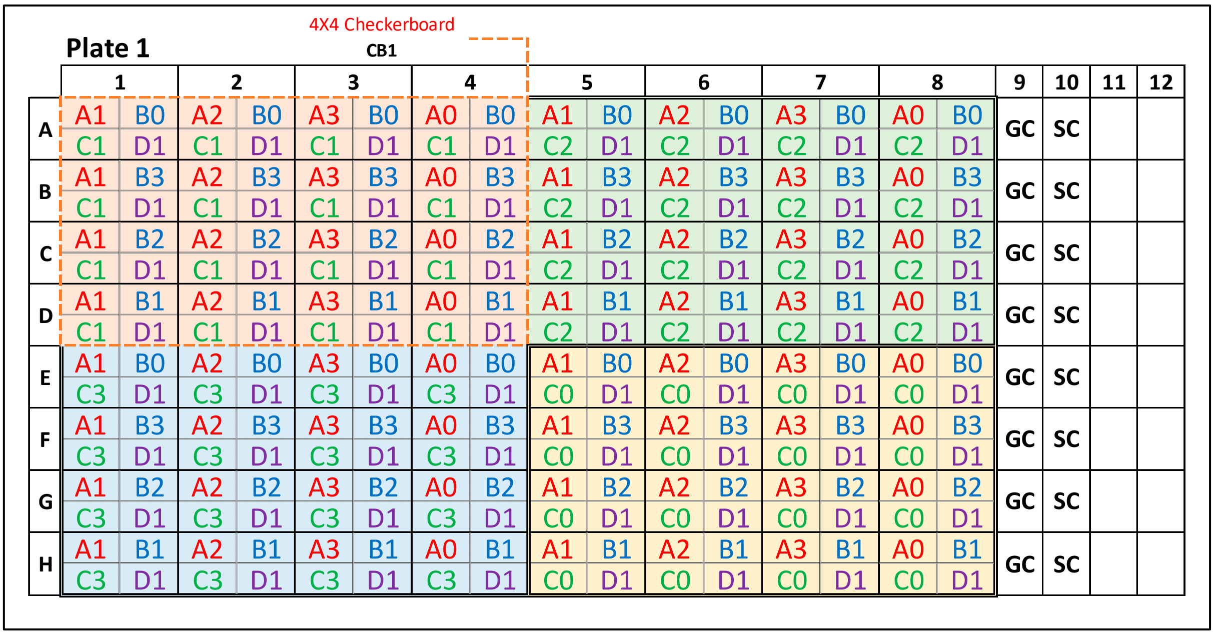

Figure S4. Drug combination Assay Final Layout: Final 96-well plate layout for one of four plates prepared for a Four-Drug combination assay showing the drug concentration of each of the four test compounds within each well. **A** refers to concentration of Drug A, **B** refers to the concentration of Drug B, **C** refers to the concentration of Drug C, **D** refers to the concentration of Drug D. Each column contains different concentration of drug A, each row has a different concentration of drug B, each 4x4 checkerboard contains different concentration of drug C, and each plate contains a different concentration of drug D, with the example plate shown having concentration 1 (D1). **GC**: Growth Control, **SC**: Sterility Control.

Table S1. Three highest Bliss scoring two-drug combinations against *E. coli*: Table showing individual concentrations (Conc 1, Conc 2), % inhibition of bacterial growth, synergy score and average synergy score against *E. coli*.

	Drug 1-Drug 2	Conc 1 (µg/mL)	Conc 2 (µg/mL)	% Inhibition	Synergy Score
E. coli		7.81	156.25	75.49	0.49
	AgNO$_3$ - Chitosan	15.63	156.25	85.56	0.40
		7.81	312.50	97.31	0.31
		6.25	8.49	67.40	0.34
	Nisin - AgNO$_3$	3.13	8.49	68.26	0.33
		1.56	8.49	64.05	0.29
		5.00	125.00	98.46	0.24
	AgNO$_3$ - ZnO	5.00	62.50	77.63	0.23
		5.00	31.25	66.38	0.21
		0.39	312.50	97.73	0.11
	Nisin - Chitosan	0.20	312.50	97.12	0.10
		0.78	312.50	96.40	0.10
		312.50	31.25	98.78	0.14
	Chitosan - ZnO	312.50	15.63	93.97	0.10
		312.50	62.50	98.79	0.09
		31.25	62.50	47.39	0.09
	Nisin - ZnO	15.63	500.00	100.00	0.07
		31.25	500.00	100.00	0.07

Table S2. Three highest Bliss scoring two-drug combinations against *S. aureus*: Table showing individual concentrations (Conc 1, Conc 2), % inhibition of bacterial growth, synergy score and average synergy score against *S. aureus*.

	Drug 1-Drug 2	Conc 1 (µg/mL)	Conc 2 (µg/mL)	% Inhibition	Synergy Score
S. aureus		15.63	156.25	69.00	0.39
	$AgNO_3$-Chitosan	7.81	156.25	62.75	0.35
		15.63	78.13	36.23	0.20
		1.56	4.25	77.46	0.27
	Nisin-$AgNO_3$	0.78	4.25	58.17	0.24
		0.78	8.49	98.98	0.20
		10.00	125.00	82.23	0.41
	$AgNO_3$-ZnO	20.00	62.50	95.47	0.20
		5.00	125.00	60.83	0.17
		3.91	156.25	50.04	0.31
	Nisin-Chitosan	1.95	78.13	18.98	0.22
		3.91	78.13	27.05	0.21
		78.13	7.81	3.05	0.11
	Chitosan-ZnO	78.13	15.63	2.60	0.10
		156.25	125.00	73.85	0.06
		0.24	31.25	14.02	0.06
	Nisin-ZnO	1.95	15.63	3.69	0.06
		0.98	15.63	6.10	0.05

Table S3. Three highest Bliss scores of each two-drug combination versus *S. epidermidis*: Table showing individual concentrations (Conc 1, Conc 2), % Inhibition of bacterial growth, synergy score and average of synergy score against *S. epidermidis*.

	Drug 1-Drug 2	Conc 1 (µg/mL)	Conc 2 (µg/mL)	% Inhibition	Synergy Score
S. epidermidis		2.50	4.88	29.61	0.31
	$AgNO_3$-Chitosan	10.00	78.13	99.08	0.22
		2.50	19.53	56.96	0.21
		1.95	10.00	78.07	0.69
	Nisin-$AgNO_3$	0.49	10.00	40.90	0.68
		0.24	10.00	39.81	0.66
		5.00	62.50	88.24	0.50
	$AgNO_3$-ZnO	10.00	62.50	95.40	0.44
		5.00	31.25	69.25	0.38
		7.81	156.25	87.28	0.11
	Nisin-Chitosan	1.95	156.25	71.93	0.01
		15.63	0.00	97.82	0.00
		4.88	15.63	22.15	0.29
	Chitosan-ZnO	9.77	15.63	31.35	0.28
		4.88	31.25	26.47	0.24
		1.95	125.00	83.63	0.16
	Nisin-ZnO	3.91	125.00	91.79	0.15
		0.24	31.25	17.39	0.10

Table S4. Three highest Bliss Synergy Scores of each three-drug combination versus *E. coli*, *S. aureus* and *S. epidermidis*. Table showing individual drug concentrations (Conc 1, Conc 2, Conc 3), % inhibition of bacterial growth, combination synergy score and average score against *E. coli*, *S. aureus* and *S. epidermidis*.

	Drug 1-Drug 2-Drug 3	Conc 1 (µg/mL)	Conc 2 (µg/mL)	Conc 3 (µg/mL)	% Inhibition	Synergy Score	Average Synergy
E. coli	Chitosan-$AgNO_3$-Nisin	80.00	4.00	3.91	99.13	0.40	0.38
		160.00	2.00	3.91	98.66	0.37	
		80.00	4.00	7.81	98.92	0.36	
	Chitosan-$AgNO_3$-ZnO	40.00	4.00	20.00	100.00	0.15	0.14
		40.00	4.00	20.00	99.55	0.14	
		80.00	0.50	20.00	97.15	0.13	
	Nisin-$AgNO_3$-ZnO	31.25	1.00	31.25	77.38	0.48	0.36
		31.25	1.00	62.50	74.84	0.31	
		31.25	0.50	62.50	58.62	0.29	
	Nisin-Chitosan-ZnO	3.91	312.50	125.00	100.00	0.21	0.20
		0.98	9.77	31.25	26.64	0.19	
		3.91	312.50	62.50	97.72	0.19	
S. aureus	Chitosan-$AgNO_3$-Nisin	78.13	8.00	0.63	99.26	0.64	0.56
		78.13	16.00	0.63	99.32	0.59	
		39.06	8.00	0.63	76.57	0.46	
	Chitosan-$AgNO_3$-ZnO	39.06	4.00	62.50	98.24	0.29	0.28
		39.06	8.00	62.50	98.32	0.29	
		78.13	8.00	62.50	97.55	0.27	
	Nisin-$AgNO_3$-ZnO	0.31	8.00	39.06	98.32	0.40	0.38
		0.63	8.00	39.06	98.39	0.40	
		1.25	8.00	39.06	100.00	0.35	
	Nisin-Chitosan-ZnO	3.91	39.06	62.50	98.66	0.93	0.83
		0.98	156.25	62.50	99.39	0.84	
		0.98	78.13	62.50	71.45	0.71	
S. epidermidis	Chitosan-$AgNO_3$-Nisin	39.06	16.00	0.63	95.12	0.51	0.43
		78.13	16.00	0.63	96.15	0.43	
		39.06	8.00	1.25	92.17	0.35	
	Chitosan-$AgNO_3$-ZnO	40.00	2.00	10.00	95.99	0.36	0.35
		20.00	4.00	10.00	87.95	0.35	
		40.00	4.00	10.00	98.40	0.32	
	Nisin-$AgNO_3$-ZnO	1.25	2.00	5.00	33.58	0.63	0.53
		1.25	4.00	5.00	46.64	0.52	
		2.50	4.00	5.00	72.31	0.45	
	Nisin-Chitosan-ZnO	0.98	39.06	31.25	99.36	0.22	0.17
		1.95	39.06	31.25	99.42	0.21	
		1.95	625.00	31.25	99.22	0.08	

Table S5. Three highest Bliss Synergy Scores and the average of each four-drug combination versus *E. coli, S. aureus* and *S. epidermidis*: Table showing individual concentrations (Conc 1, Conc 2, Conc 3, Conc 4), % Inhibition of bacterial growth, synergy score and average synergy score of the three against *E. coli*, *S. aureus* and *S. epidermidis*.

	Drug 1-Drug 2-Drug 3-Drug 4	Conc 1 (µg/mL)	Conc 2 (µg/mL)	Conc 3 (µg/mL)	Conc 4 (µg/mL)	% Inhibition	Synergy Score	Average Synergy
E. coli	Chitosan-Nisin-$AgNO_3$-ZnO	80.00	31.25	8.00	40.00	98.90	0.44	0.36
		80.00	1.95	8.00	40.00	76.61	0.34	
		80.00	1.95	8.00	10.00	69.13	0.30	
S. aureus	Chitosan-Nisin-$AgNO_3$-ZnO	78.13	0.39	4.00	62.50	98.15	1.04	0.91
		19.53	0.39	4.00	62.50	85.02	0.85	
		78.13	1.56	4.00	62.50	98.52	0.85	
S. epidermidis	Chitosan-Nisin-$AgNO_3$-ZnO	20.00	1.25	8.00	10.00	24.96	1.30	1.11
		20.00	1.25	8.00	40.00	99.12	1.13	
		80.00	1.25	8.00	10.00	98.77	0.90	
